# A Bacterial Quorum Sensing Molecule Elicits a General Stress Response in *Saccharomyces cerevisiae*

**DOI:** 10.3389/fmicb.2021.632658

**Published:** 2021-09-16

**Authors:** Antonia Delago, Rachel Gregor, Luba Dubinsky, Rambabu Dandela, Adi Hendler, Pnina Krief, Josep Rayo, Amir Aharoni, Michael M. Meijler

**Affiliations:** ^1^Department of Chemistry, Ben-Gurion University of the Negev, Be’er Sheva, Israel; ^2^The National Institute for Biotechnology in the Negev, Ben-Gurion University of the Negev, Be’er Sheva, Israel; ^3^Department of Life Sciences, Ben-Gurion University of the Negev, Be’er Sheva, Israel

**Keywords:** quorum sensing, interkingdom communication, *Saccharomyces cerevisiae*, *Pseudomonas aeruginosa*, stress response, Msn 2/4, N-acyl homoserine lactones

## Abstract

Bacteria assess their population density through a chemical communication mechanism termed quorum sensing, in order to coordinate group behavior. Most research on quorum sensing has focused primarily on its role as an intraspecies chemical signaling mechanism that enables the regulation of certain phenotypes through targeted gene expression. However, in recent years several seminal studies have revealed important phenomena in which quorum sensing molecules appear to serve additional roles as interspecies signals that may regulate microbial ecology. In this study, we asked whether the budding yeast *Saccharomyces cerevisiae* can sense chemical signals from prokaryotes. When exposed to a variety of quorum sensing molecules from different bacterial species and from *Candida albicans* we found that *N*-(3-oxododecanoyl)-L-homoserine lactone (C12) from the opportunistic human pathogen *Pseudomonas aeruginosa* induces a remarkable stress response in yeast. Microarray experiments confirmed and aided in interpreting these findings, showing a unique and specific expression pattern that differed significantly from the response to previously described stress factors. We further characterized this response and report preliminary findings on the molecular basis for the recognition of C12 by the yeast.

## Introduction

Quorum sensing (QS) is a cell-density-dependent communication mechanism used by unicellular organisms to coordinate population-wide gene expression that is of benefit to the group as a whole. It is based on the release and detection of small diffusible signal molecules called autoinducers (AIs). AIs are secreted by individual cells at a given rate during the growth of a population. When the group reaches a threshold cell density and AI concentration, a cascade of gene expression and protein transcription is set in motion, resulting in increased signal and receptor expression that enables a feedback loop to control cell-density-dependent phenotypes and processes (Popat et al., 2015). For example, the sophisticated QS system of *Pseudomonas aeruginosa* has been studied extensively due to the frequent nosocomial infections that this opportunistic pathogen causes, especially in patients who are immunocompromised or suffer from cystic fibrosis. *P. aeruginosa* has several QS systems which are organized in a hierarchical manner (Lee and Zhang, 2015). On top of this hierarchy is the LasI/LasR system with its AI *N*-(3-oxododecanoyl)-L-homoserine lactone (C12). This system co-regulates the RhlI/RhlR system with its AI *N*-butyryl-homoserine lactone (C4). The third main *P. aeruginosa* QS system is based on the *Pseudomonas* quinolone signal (PQS), a multifaceted QSM (Cugini et al., 2007, 2010) responsible for the expression of several virulence factors (Haussler and Becker, 2008; Maura et al., 2016). The PQS system is intricately connected with the LasI/LasR and the RhlI/RhlR systems and plays an important role in linking these two systems together (Rampioni et al., 2016).

QS exists among eukaryotes as well: It has been demonstrated that the budding yeast *Saccharomyces cerevisiae* uses small aromatic alcohols, such as tryptophol and phenylethanol, to control the formation of pseudohyphae, a trait associated with multicellular processes such as biofilm formation (Reynolds and Fink, 2001). The pathogenic yeast *Candida albicans* utilizes a small acyclic sesquiterpene alcohol, farnesol, as a QSM (Hornby et al., 2001; Lindsay et al., 2012). While *C. albicans* has been shown to also produce tryptophol and phenylethanol (Lingappa et al., 1969; Hazen and Cutler, 1979; Chen and Fink, 2006; Hogan, 2006), these compounds do not promote the *C. albicans* yeast-to-hyphae transition, but rather have been shown to repress hyphal development (Chen and Fink, 2006).

In the past decades QS has mostly been studied as a phenomenon that benefits a particular species in question. However, in recent years it is becoming increasingly evident that other species are able to sense and respond to the secreted signals as well, and that such signals may govern microbial ecology in a given environment (Kravchenko et al., 2008; Jarosz et al., 2011; Teplitski et al., 2011). One such interaction is that between *P. aeruginosa* and *C. albicans*. For example, the *P. aeruginosa* QSM C12 has been shown to inhibit *C. albicans* filamentation (Hogan et al., 2004) via the Ras-cyclic AMP-dependent protein kinase A (cAMP/PKA) pathway, mimicking the effect of *Candida*’s native QSM farnesol (Davis-Hanna et al., 2008; Hall et al., 2011). In *P. aeruginosa*, the *C. albicans* QSM farnesol, in return, inhibits synthesis of the *Pseudomonas* quinolone signal (PQS) (Cugini et al., 2007). *C. albicans* and *P. aeruginosa* are both opportunistic human pathogens able to take advantage of weaknesses in the human immune system, and they coexist in infections such as in the lungs of cystic fibrosis patients (Gibson et al., 2009). In general, recent studies have shown that chemical signaling between bacteria and eukaryotes is a crucial factor in determining the formation of intricate relationships between species in a given environment. Prime examples are the recently revealed interactions between *P. aeruginosa* and *Aspergillus fumigatus* (Moree et al., 2012) and the remarkable effects induced by *Algoriphagus machipongonensis* and *Vibrio fischeri* on the multicellular development and mating behavior of the choanoflagellate *Salpingoeca rosetta*, the closest living relative of animals (Alegado et al., 2012; Woznica et al., 2017).

In this study, we set out to examine potential interactions between *S. cerevisiae* and *P. aeruginosa*, both ubiquitous microbes that have been isolated from a wide range of plant and soil niches across the globe (Green et al., 1974; Fay and Benavides, 2005). In general, little has been published on interactions between *P. aeruginosa* and fungal species other than *C. albicans*. The molecular basis of such interactions may be similar to what is known regarding *P. aeruginosa* and *C. albicans* or proceed by other mechanisms entirely. We chose to begin with *S. cerevisae* as it is a well-studied model organism that offers an abundance of genetic tools to study interactions between bacterial and fungal cells.

One field of research for which *S. cerevisiae* is an important model system is the study of cellular stress response. Yeast cells have evolved to survive sudden, often drastic and stressful changes in their environment and a variety of such stressful conditions have already been thoroughly experimentally characterized, including growth temperature, osmotic concentration and ionic strength of the growth medium, exposure to toxins, starvation for a variety of nutrients, irradiation, and desiccation (Attfield et al., 1997). In response to a sudden shift in conditions, yeast cells initiate a multifaceted response that involves an often-transient arrest of normal cellular processes. Cellular response mechanisms include environmental sensing and signal transduction pathways that lead to significant alterations in gene expression programs. Such induction or repression of gene expression under stress conditions enables fast adaptation to diverse conditions, resulting in increased cell fitness and survival (Gasch et al., 2000). To the best of our knowledge, no report has been published on a potential stress response in yeast to QSMs from bacteria or fungi.

In the present study we show that *S. cerevisiae*, like *C. albicans*, responds to the presence of *P. aeruginosa*. We demonstrate a general stress response of *S. cerevisiae* that is specific to the *Pseudomonas* AI C12. We further present data that provide important clues on the role of this signaling molecule for interkingdom communication that could govern the ecology of these microbes in a given environment.

## Materials and Methods

### Strains and Cultivation Conditions

*S. cerevisiae* strains were grown at 30°C for 48 h on agar plates containing standard YPD or Selective Complete medium (SC, -His). Plated colonies were stored at 4°C. Yeast strains used in this study were: W303 WT, W303 Hsp12-GFP, W303 Δ*msn2* Hsp12-GFP, W303 Δ*msn4* Hsp12-GFP, W303 Δ*msn2*Δ*msn4* Hsp12-GFP; MatA WT; BY4741 his3Δ1:KanMX. *Pseudomonas aeruginosa* wild-type strain PAO1-UW was grown at 37°C for 18–24 h on LB agar. Overnight liquid cultures were prepared in LB medium without antibiotics; overnight cultures of PAO-JP2 (Δ*lasI*Δ*rhlI*) were prepared in LB medium containing 300 μg/mL trimethoprim (TMP).

### Compounds

A panel of quorum sensing compounds were purchased from Sigma-Aldrich, Acros or synthesized as previously reported: *N*-(3-oxododecanoyl)-L-homoserine lactone (C12) (Kravchenko et al., 2008), *N*-butyryl-homoserine lactone (C4), *N*-octanoyl-L-homoserine lactone (C8), 4,5-dihydroxy-2,3-pentanedione (DPD) (Mandabi et al., 2015), and farnesol. Control compounds were the unnatural *R* enantiomer of C12, *N*-(3-oxododecanoyl)-D-homoserine lactone (*R*-C12), and the lactam analog of C12 (S)-3-oxo-N-(2-oxopyrrolidin-3-yl)dodecanamide (C12-lactam) (Kravchenko et al., 2006). For receptor labeling experiments, an alkyne-appended diazirine analog of C12 (P6) was synthesized as previously reported by us (Dubinsky et al., 2009).

### FACS Analysis of Hsp12-GFP Expression

The above panel of QS molecules was incubated with *S. cerevisiae* cultures labeled with Hsp-12 GFP reporters in order to screen for the induction of a stress response. In brief, *S. cerevisiae* overnight cultures were diluted to OD_600_ ∼0.1 and grown at 30°C until reaching the exponential growth phase (OD_600_ ∼0.4–0.6). For FACS measurement 500 μL of yeast culture were transferred into 1.5 mL centrifuge tubes and compounds were added at the indicated concentrations. Cells were briefly vortexed and then incubated at room temperature for 40–45 min. Samples were analyzed by flow cytometry (FACS Calibur, BD Biosciences, Franklin Lakes, NJ), and the Hsp12-GFP fluorescence intensity of 10,000 yeast cells was measured. For heat shock controls 500 μL of cell culture was transferred into 1.5 mL centrifuge tubes and incubated at 37°C for 40–45 min. Data acquired from FACS measurements were imported into FlowJo Single Cell Analysis System (FlowJo, LLC, OR, United States), which allowed direct calculation of median, mean and standard error. From FlowJo data were directly exported to Excel for graphical presentation.

### Microarray Analysis of Gene Expression

Samples were analyzed by microarray in order to determine the effects of C12 on the yeast transcriptome. First, 1.5 mL of yeast culture *S. cerevisiae* W303 Hsp12-GFP (OD_600_ ∼0.4–0.6) was transferred into centrifuge tubes and test compound solutions were added. Treatments used were DMSO (control), 100 μM C12, 100 μM C12-lactam, and 300 μM H_2_O_2_. Cells were briefly vortexed and incubated at 30°C for 30–35 min, after which 1,000 μL were transferred into fresh tubes and cells were pelleted by centrifugation. The supernatant was discarded and the pellet immediately snap-frozen in liquid nitrogen. The remaining 500 μL were analyzed by FACS for Hsp-12-GFP expression levels in order to validate stress response before performing the microarray experiment. Total RNA was extracted from the frozen pellets using a QuickExtract^TM^ RNA extraction kit (Epicenter, Madison, WI). Extracted total yeast RNA was then checked for integrity using an Affimetrix Bioanalyzer. The microarray experiments were performed using GeneChip Yeast 2.0 Array (Affymetrix, Santa Clara, CA). Sample preparation (50 ng/μL total RNA) was performed according to the protocol provided with the chips.

### Microarray Data Analysis

The microarray results were filtered based on threshold intensity, in which probe sets without at least one sample with an intensity value greater or equal to 5 (log2 scale) were discarded, resulting in a total of 4,612 probe sets. A one-way ANOVA for the treatment effect was applied, with the following groups compared: C12 vs. C12-lactam; H_2_O_2_ vs. C12-lactam; DMSO (solvent control) vs. C12-lactam; and C12 vs. H_2_O_2_, with a cutoff of FDR < 0.05 and F_*C*_ > 1.3 or < –1.3. The data was visualized by principal coordinates analysis (PCA) and by hierarchical clustering using Pearson dissimilarity, and one of the replicates for the C12 treatments was excluded as an outlier and removed from analysis (Supplementary Figure 1). After data analysis, resulting gene lists were uploaded onto YeastCyc BIOCHEMICAL PATHWAYS^1^ and FunSpec^2^ online analysis tools to extract biochemical pathways that are affected by the different stressors.

### Co-culture Experiments Between *S. cerevisiae* and *P. aeruginosa*

For liquid co-culture experiments, yeast and *Pseudomonas* overnight starters were diluted to OD_600_ ∼ 0.01. Diluted yeast and bacterial cultures were then mixed 1:1 and incubated at 30°C for 10–96 h. Yeast culture diluted 1:1 with LB medium served as control. At various time points (7, 24, and 48 h) co-cultures and controls were diluted in sterile DDW and plated on selective YPD agar containing 150 μg/mL tetracycline (Tet)—allowing the yeast to grow but inhibiting the growth of the bacteria.

### Viability Assays

A single colony of *S. cerevisiae* strain BY4741 was inoculated in liquid YPD medium and grown with agitation for 4 h at 30°C. Then, aliquots were incubated with 50 μM C12 or DMSO control for 30 min., followed by 10 min. of extreme oxidative stress with 0.1% H_2_O_2_. Then, samples were diluted 1:100 and 60 μL each were plated onto YPD agar. After 96 h at 30°C, the number of colony forming units (cfu) on each plate was counted and analyzed.

### Receptor Labeling Using Diazirine Alkyne C12 Analog (P6)

Overnight cultures in YPD liquid medium were diluted into YPD to an OD_600_ ∼0.1 and incubated at 30°C until they reached OD_600_ ∼0.4–0.6. The yeast culture was then divided into aliquots in 50 mL Falcon tubes wrapped in aluminum foil for light protection. To each yeast culture we added test compounds (100 μM probe or 50 μM probe with 100 μM C12) or control solutions (10 μM lysozyme-alkyne; DMSO (0.5%); 100 μM C12). The tubes were vortexed briefly and then incubated for 30–35 min at RT. Following incubation each treatment group was transferred into a separate petri dish and irradiated at 365 nm for 10 min using a UV table. Immediately after, the yeast cultures were pelleted by centrifugation for 2 min at 4,000 rpm at 4°C, the supernatant decanted, and the pellet snap-frozen in liquid nitrogen. Then, 4 μL protein inhibitor (Sigma) and 400 μL CelLytic^TM^ Y Cell Lysis Reagent (Sigma-Aldrich Israel Ltd., Rehovot, Israel) were added and cells were lysed by rigorous mixing for 30 min. The lysates were then centrifuged for 25 min at 4,000 rpm at 4°C, and the supernatant was transferred into fresh 1.5 mL centrifuge tubes and kept at 4°C or stored at –20°C until further processing by CuAAC chemistry to rhodamine azide for in-gel visualization.

### CuAAC Chemistry Using Rhodamine Azide (RhN_3_)

The protein concentration of each sample was measured using a BCA protein assay (Pierce^®^BCA Protein Assay Kit; THERMO Scientific, Rockford, IL). According to the sample with the lowest protein concentration samples were diluted in PBS. Click chemistry was done with a total volume of 100 μL. To 89 μL sample we added 50 μM RhN_3_ (from a 50 mM stock in DMSO), 1 mM tris (2-carboxyethyl)phosphine (TCEP; from a 50 mM solution prepared fresh every time in DDW), 100 μM Tris[(1-benzyl-1H-1,2,3-triazol-4-yl)methyl]amine (TBTA, from a 1.7 mM stock in 1:5 DMSO:t-butanol). Samples were briefly mixed before adding 1 mM CuSO_4_ (from a 50 mM stock in DDW). After a final brief mix, samples were incubated overnight at room temperature under constant agitation.

For visualization, samples were then prepared for SDS polyacrylamide gel electrophoretic separation. To each sample we added gel loading buffer and reducing agent. The samples were heated to 70°C for 10 min before loading them onto a NuPAGE^®^ Bis-Tris 12% Precast Gel (Invitrogen Corp., Grand Island, NY). For molecular weight determination a Precision Plus Protein Prestained Dual color Standard (Bio-Rad Laboratories Inc.) was run in parallel to our samples. After electrophoresis, performed under light protection, RhN_3_ labeled proteins were visualized with a Fujifilm LAS-3000 Imager.

### Initial Chemical Proteomics Experiments

To affinity purify and characterize proteins that covalently bound the C12 probe, we incubated samples as described for the in-gel fluorescence experiments. However, click chemistry was performed using an Azide-PEG3-Biotin Conjugate (Jena Bioscience GmbH, Jena, Germany). The total volume used for the click reaction was 1 mL. To 870 μL sample we added 150 μM Biotin-N_3_ (from a 5 mM stock in DDW), 1 mM TCEP, and 100 μM TBTA. Samples were briefly mixed before adding 1 mM CuSO_4_. After a final brief vortex, samples were incubated ON at RT under constant agitation. For affinity purification for each sample 200 μL of streptavidin agarose resin (Invitrogen Corp., Grand Island, NY) were transferred into 0.8 mL centrifuge columns (Pierce Biotechnology, Rockford, IL) which were placed into 1.5 mL centrifuge tubes. The resin was washed 5 times with PBS before it was transferred into the click solution. Click solution and beads were incubated for 90–120 min at RT under constant gentle end-over-end rotation. After incubation samples were transferred back into the centrifuge columns and gently spun down. The run through was collected and the agarose beads washed 4 times with 350 μL PBS each—collecting each run through in a separate tube. Then the beads were incubated with 350 μL 0.1% SDS in PBS for 10 min at RT under constant gentle rotation. Thereafter the beads were washed three times with 350 μL PBS each, followed by three washes with 350 μL DDW each. For Orbitrap analysis, samples were eluted by adding 300 μL 8 M Guanidine HCl, heating them for 10 min at 60°C, then washing them once with 300 μL PBS, before adding another 300 μL 8 M Guanidine HCl and heating them to 90°C for 10 min. All eluted samples were transferred into separate 500 μL Amicon Ultra Centrifugal Filters (10 kDa cut-off; Milipore Corp., Billerica, MA) to concentrate them ∼50 times (to about 30–40 μL). Concentrated elutes were collected in low protein binding centrifuge tubes and stored at –20°C. Controls were performed in which either no probe or lysozyme modified with alkyne groups was added to cell lysates before the click reaction and affinity purification. All samples were analyzed (LC-MS/MS—Thermo-Finnigan Orbitrap) by the Smoler Proteomics Center [Israeli Institute of Technology (Technion), Haifa, Israel].

## Results

### 3-Oxo-C12-Homoserine Lactone (C12) Induces a Specific Stress Response in Yeast

**FIGURE 1:**
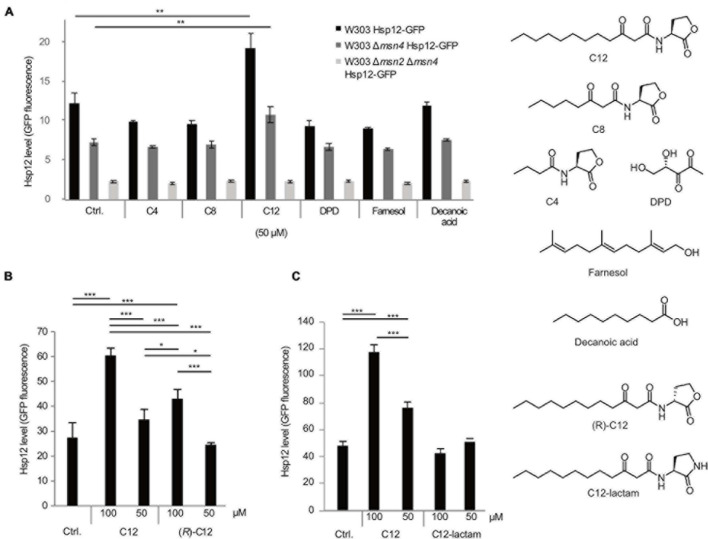
Selective response of *S. cerevisiae* cells to different QSMs. Yeast cells (W303 WT, black bars, Δ*msn4* mutant, dark gray bars, and Δ*msn2*Δ*msn4* double mutant, light gray bars) were exposed to different QSMs for 45 min and the stress response measured by expression levels of GFP-labeled small heat shock protein Hsp12. **(A)** Initial screening for stress response to different QSMs and controls, tested at 50 μM concentration: C12: 3-oxo-C12 HSL; C8: 3-oxo-C8 HSL; C4: C4 HSL; DPD: 4,5-dihydroxy-2,3 pentanedione; farnesol; decanoic acid. Ctrl. = solvent control (DMSO). C12 induced a significantly higher stress response than the control. Results are presented as the median of 10,000 cells for duplicate samples (± standard deviation) and are representative of other experiments. **(B)** Additional experiments were performed to determine the specificity of the response to C12. The stress response to C12 showed a concentration-dependent effect. The unnatural enantiomer of C12 induced a lower stress response than C12. **(C)** An additional control compound, C12-lactam, did not induce a significant stress response. For all panels, results are presented as the median of 10,000 cells for triplicate samples (± standard deviation) and are representative of other experiments. Significance of the variance between groups was calculated using a one-way ANOVA test, followed by pairwise comparisons using Tukey multiple comparison of means. Key: *p ≤ 0.05, **p ≤ 0.01, ***p ≤ 0.001.

*S. cerevisiae* cells were treated with a diverse panel of QSMs from different bacteria in order to measure the yeast stress response (Figure 1A). The stress response was quantified by the expression of the small heat shock protein Hsp12, labeled with green fluorescent protein (GFP), a system used previously to examine expression and the role of Msn2/4 transcription factors (TF) involved in the general yeast stress response (Sadeh et al., 2011, 2012). Only cells exposed to the *Pseudomonas* QSM C12 showed a significant stress response (Figure 1A, black bars). Other QSMs tested, including C4 HSL (*P. aeruginosa* RhlI/RhlR system), *N*-octanoyl-L-homoserine lactone (3-oxo-C8 HSL; *Agrobacterium tumefaciens* TraI/TraR system), 4,5-dihydroxy-2,3-pentanedione (DPD), and the *C. albicans* QSM farnesol did not induce a stress response above control levels (Figure 1A, black bars). Decanoic acid was included as a lipophilic control, and also did not induce a stress response above control levels (Figure 1A, black bars). After initially testing a range of concentrations, we found that the 50–100 μM range elicited the most reproducible Hsp12 signal, and showed no inhibitory effects on growth. While these concentrations are substantially higher than the nanomolar concentrations that activate the cognate receptor in P. aeruginosa (Pearson et al., 1994) and other bacteria (Pinto and Winans, 2009; de Almeida et al., 2018), they are comparable to those used in previous interkingdom studies (Chhabra et al., 2003; Kravchenko et al., 2008; Karlsson et al., 2012; Maurer et al., 2015; Rayo et al., 2021). Future studies on the mechanism of this stress response are needed to determine a more precise active concentration range.

To confirm that the increase in Hsp12-GFP expression is an Msn2/4 mediated stress response, we repeated the screen on strains deleted in either one or both of the Hsp12 transcription factors. The stress response was reduced in the Msn4 mutant (Figure 1A, dark gray bars) and completely abolished in the double deletion strain (Figure 1A, light gray bars), indicating that this effect is indeed mediated via the Msn2/4 pathway.

We proceeded with further control experiments to confirm that the yeast response was truly specific to C12. We tested the non-natural (*R*)-enantiomer of C12, (*R*)-C12, which is inactive as a quorum sensing signal in *P. aeruginosa* (Figure 1B). (*R*)-C12 triggered a less strong stress response than that of the natural enantiomer. An additional control compound, C12-lactam, in which the lactone ring is replaced by a lactam ring, did not induce a significant stress response (Figure 1C).

### mRNA Microarray Analysis

**FIGURE 2:**
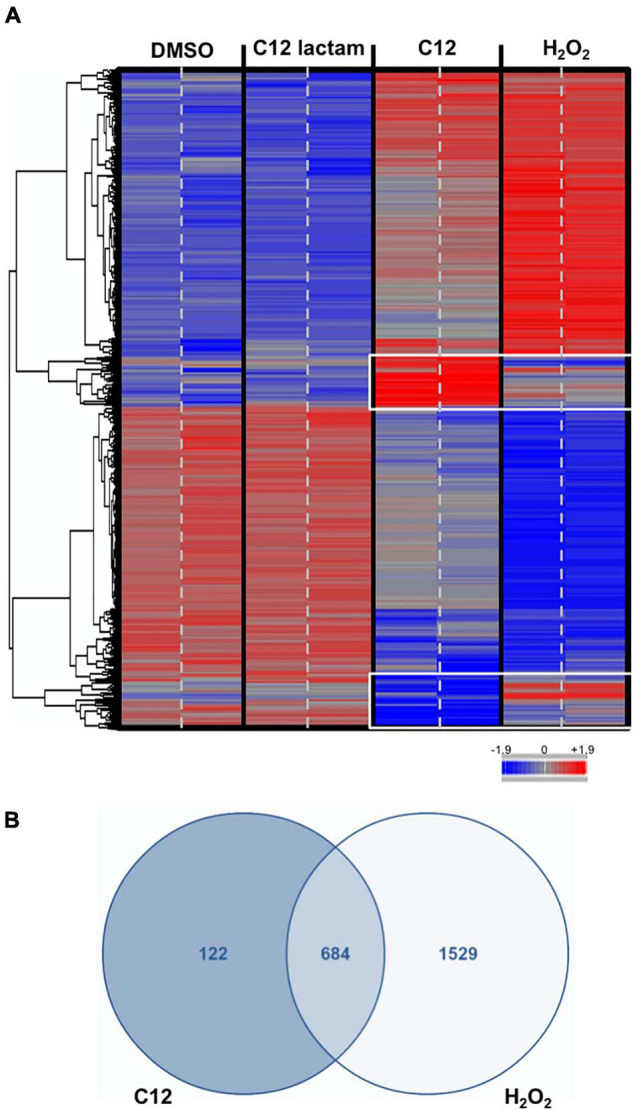
Microarray analysis reveals changes in gene expression specific to C12. **(A)**. The heatmap shows the gene expression profile after exposure to controls (DMSO control, C12-lactam and oxidative stress) and C12. Down-regulation shown in blue, up-regulation shown in red. The expression profile after exposure to C12 is clearly different from the controls. Oxidative stress and C12 show a similar general trend in gene expression, but several expression changes are unique to C12 (white frame). The microarray results from two chips for each treatment are shown; samples were measured in triplicate, but one replicate was excluded (see Supplementary Figure 1). **(B)**. Of the 806 genes that underwent expression changes due to C12 treatment, 122 (15%) were unique to C12, whereas 684 were shared.

In order to further explore the C12-induced stress response, microarray assays were performed. Yeast samples treated with 100 μM of C12 were chosen for microarray analysis, as well as 100 μM of C12-lactam and a solvent DMSO control (samples chosen are shown in Figure 1C). Additional samples were treated with 0.3 mM hydrogen peroxide, for comparison to the oxidative stress response, as has been reported previously (Gasch et al., 2000). Microarray analysis revealed that the expression pattern in response to C12 is distinctly different from both the controls (Figure 2A and Supplementary Figure 1). The control C12-lactam shows an expression pattern similar to the solvent control. The expression profile after exposure to 0.3 mM H_2_O_2_ is similar to what has been reported previously by Gasch et al. (2000) (Supplementary Figure 2). A direct comparison between C12 treatment and oxidative stress reveals similar trends, with some key differences (Figure 2A). Both induced the regulation of a number of antioxidants and proteins involved in carbon metabolism, resulting in metabolic redirection from glycolysis to the pentose phosphate pathway. Other pathways upregulated by both hydrogen peroxide and by C12 include genes involved in protein degradation, the synthesis of heat shock proteins and chaperones. Pathways involved in DNA replication, on the other hand, were downregulated. However, of the 806 genes up- or down-regulated in response to C12 treatment, 122 (15%) were specific to C12 (Figure 2B). Out of the genes found to be uniquely affected by C12, a number are involved in lipid synthesis and transport, including a gene upregulated 20-fold, *RSB1*. Additionally, C12 treatment induced the downregulation of several genes involved in iron transport, assimilation and homeostasis (*FTR1*, *FET3*, *SIT1*, *FRE1*). In contrast, these same genes are upregulated upon exposure to hydrogen peroxide, indicating that this response pattern is distinct from the oxidative stress response.

### *S. cerevisiae* Shows Reduced Viability in the Presence of *P. aeruginosa*

**FIGURE 3:**
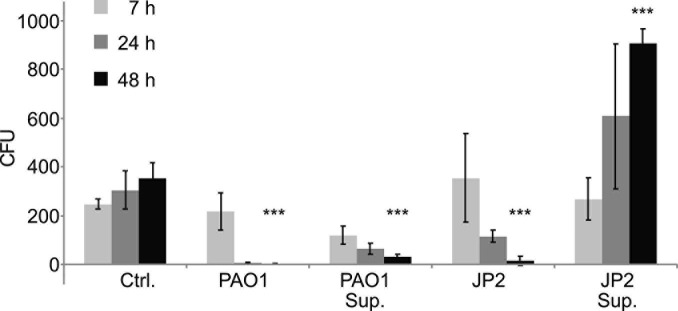
Liquid co-cultures of wild type yeast with *Pseudomonas* reveal growth inhibitory effect of *Pseudomonas* supernatant. Overnight cultures of *S. cerevisiae* wild-type strain W303 and *P. aeruginosa* (wild-type strain PAO1 or the Δ*rhlI*Δ*lasI* mutant PAO-JP2) were both adjusted to OD_600_∼0.01 and incubated together at 30°C under constant agitation. Additionally, *S. cerevisiae* was grown under the same conditions in the presence of PAO1 or PAO-JP2 supernatant. After 7, 24 and 48 h samples were taken, diluted in DDW and plated on selective YPD agar containing 50 μg/mL gentamicin (in order to inhibit bacterial growth and thus monitor only yeast growth/viability). Yeast viability was reduced in the presence of the bacteria for both the wild-type and the mutant, presumably due to competition for resources. However, only the supernatant of PAO1 inhibited yeast growth, indicating inhibition due to bacterial virulence factors or other QS-regulated secondary metabolites. Values are averages of three experiments (± standard deviation). The significance of the variance between groups was calculated using a one-way ANOVA test, followed by pairwise comparisons using Tukey multiple comparison of means. Key: ***p ≤ 0.001.

To examine whether the presence of *P. aeruginosa* affects the growth of *S. cerevisiae*, the two strains were grown together in liquid co-culture. The presence of the bacteria significantly reduced the number of viable yeast cells after 48 h in liquid co-culture (Figure 3). We observed a similar result when the yeast was cultured in the presence of *Pseudomonas* supernatant. The latter indicates that the growth inhibitory effect may be caused by the secretion of virulence factors by *P. aeruginosa*. When the yeast was cultured in the presence of PAO-JP2, a mutant deficient in LasI and RhlI, i.e., that cannot synthesize C12 or C4 and produces fewer QS-regulated virulence factors, we also saw a reduction in viable yeast cells. However, in the presence of only bacterial supernatant from JP2, the yeast actually showed a significant increase in growth after 48 h (Figure 3).

### Rescue Effect of C12 on Yeast Response to Oxidative Stress

**FIGURE 4:**
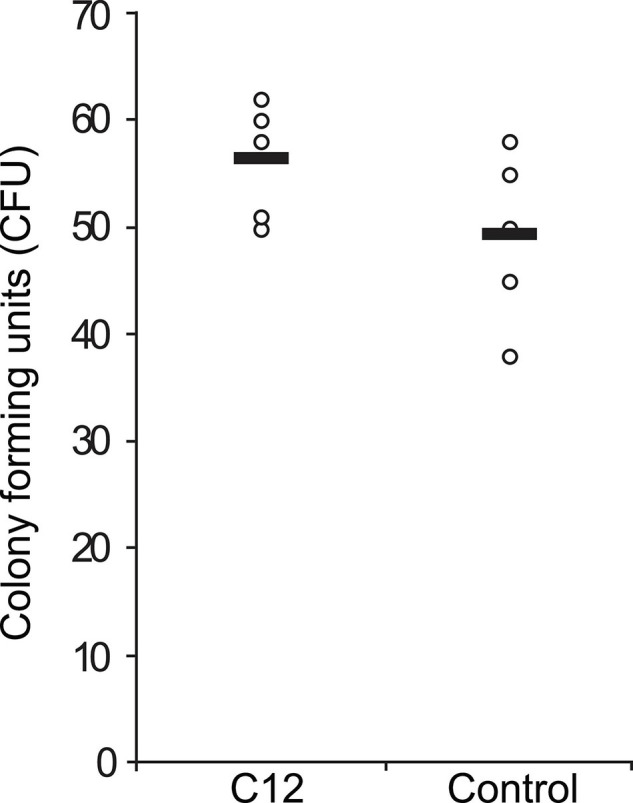
Effect of C12 on yeast response to oxidative stress. A single colony of *S. cerevisiae* strain BY4741 was inoculated in liquid YPD medium and grown with agitation for 4 h at 30°C. Then, aliquots of this dilution were pre-incubated with 50 μM C12 for 30 min and thereafter exposed to 0.1% H_2_O_2_ for 10 min. After exposure to oxidative stress the cells were diluted 1:100 in YPD medium and plated on YPD agar. After 96 h, the number of colony forming units (CFU) was counted as a measure of viable cells and compared between treatments. CFU counts for replicate samples are represented by circles (*n* = 5), and black bars indicate the mean of each group.

Next, we asked if C12 induced a rescue effect based on the environmental stress response (ESR) (Gasch, 2007), in which a mild stressor leads to higher survival rates for a subsequent, severe stress. This effect has been previously shown in *C. albicans* with its native QSM farnesol, via the cAMP/PKA pathway (Westwater et al., 2005; Deveau et al., 2010). Viability assays were performed using CFU counts on agar plates after treatment of the yeast with test or control compounds. Yeast cells in liquid YPD medium were pre-incubated with 50 μM C12 for 30 min and thereafter exposed to 0.1% H_2_O_2_ for 10 min. This concentration of hydrogen peroxide results in an approximately 50% reduction in viability (Supplementary Figure 3), and is equivalent to 32.6 mM, ∼100 times higher than the concentration used as a mild stressor in the microarray experiments. The samples were then diluted and plated on YPD agar. After 96 h, the number of colony forming units (CFU) was counted as a measure of viable cells and compared between treatments. In *S. cerevisiae* wild-type strain BY4741, pre-treatment with C12 seems to have slightly increased resistance to oxidative stress on average, although the difference was not significant (*p* = 0.07, one-tailed *t*-test) (Figure 4). However, in another wild-type strain, W303, we did not observe a difference in the number of viable yeast cells dependent on pre-treatment with C12, suggesting that this effect is strain-specific (data not shown).

### Labeling Yeast Receptors for C12

**FIGURE 5:**
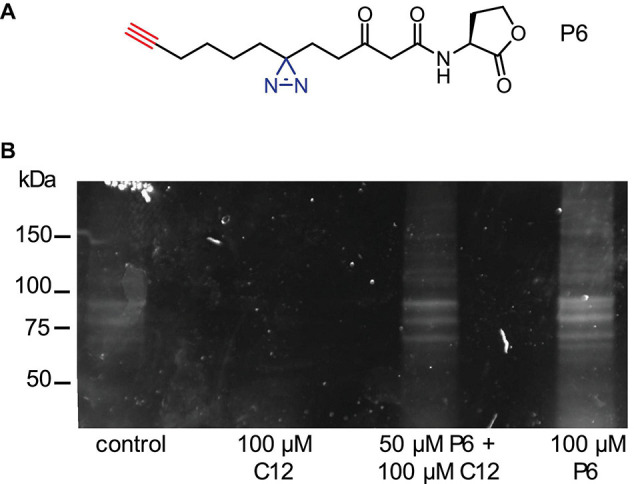
Using a reactive C12 analog probe to label putative C12 receptors in yeast. **(A)** Structures of C12 analog probe (P6) with small diazirine (blue) and alkyne (red) handles incorporated in order to uncover potential C12-receptor interactions. **(B)** Labeling experiment. Wild-type *S. cerevisiae* W303 was incubated for 30 min. with the following treatments: control solutions (DMSO; 100 μM C12), or two treatments of P6: 50 μM P6 with 100 μM C12, or 100 μM P6. The cultures were irradiated at 365 nm for 10 min on a UV table to activate covalent binding on the diazirine position to any interacting proteins. Then, cells were lysed and the lysate reacted with rhodamine azide using CuAAC chemistry, resulting in a rhodamine-labeled probe-protein complex. Labeled proteins were visualized using in-gel fluorescence.

In order to investigate the mechanism behind the *S. cerevisiae* response to C12 and activation of the Msn2/4 mediated stress response, we performed experiments to label putative receptors for this compound. Using an activity-based protein profiling (ABPP) approach (Speers and Cravatt, 2009), we synthesized an alkyne-appended diazirine analog of C12 (P6; Figure 5A), currently used by us to identify C12-binding proteins in other eukaryotes (Dubinsky et al., 2009). In short, activation of the diazirine moiety through photo-irradiation with UV-light (365 nm) induces covalent binding between the probe and proteins in its vicinity. Then, via a copper-mediated azide-alkyne cycloaddition reaction (CuAAC), the alkyne-appended proteins can be coupled to rhodamine azide, enabling the visualization of labeled proteins using in-gel fluorescence. In fluorescence labeling experiments with wild-type *S. cerevisiae* strain W303, a number of abundant proteins in the molecular weight range of ∼60–100 kDa were labeled, as well as some less abundant proteins with lower molecular weights (Figure 5B).

Next, we purified proteins covalently bound to our alkyne probe and performed mass spectrometry analysis, resulting in a number of hits that showed a strong ratio between scores obtained for the probe and for the control labeling experiments (Table 1; full data available in Supplementary Table 2). We selected the HYP2 isoform of the eukaryotic elongation initiation factor 5A (eIF-5A) for further validation. This 17 kDa protein is highly conserved among eukaryotes and has previously been linked to stress responses (Takeuchi et al., 2002; Li et al., 2010). However, initial attempts to purify recombinant eIF-5A to perform direct binding assays proved problematic due to the instability of the protein, and possibly because we only expressed one of the two isoforms, that was additionally lacking a hypusination modification. We are currently engaged in additional approaches to further study the function and role of C12 and eIF-5A in yeast.

**TABLE 1:** A list of proteins identified using chemical proteomics experiments with probe P6.

**SGD**	**Protein**	**Description**
YEL034W	HYP2	Translation elongation factor eIF-5A
YJL076W	NET1	Core subunit of the RENT complex
YHR193C	EGD2	Alpha subunit of the nascent polypeptide-associated complex (NAC)
YFR033C	QCR6	Subunit 6 of the ubiquinol cytochrome-c reductase complex
YPL022W	RAD1	Single-stranded DNA endonuclease (with Rad10p)
YLR075W	RPL10	Ribosomal 60S subunit protein L10
YLR262C-A	TMA7	Protein of unknown function, associates with ribosomes
YLR363W-A	—	Protein of unknown function, colocalizes with nucleus

*Mass spectrometry analysis of proteins covalently bound to our alkyne probe yielded a number of hits that showed a strong ratio between scores obtained for the probe and for the control labeling experiments.*

## Discussion

In the present paper we show that the budding yeast *S. cerevisiae* responds to the presence of the Gram-negative bacterium *P. aeruginosa*, or more precisely to its primary QSM C12. We provide strong evidence that the observed effect is a general stress response mediated by the transcription factors Msn2/4. Examining changes in the expression of the GFP-labeled small heat shock protein Hsp12, we measured the physiological response of *S. cerevisiae* to different bacterial QSMs. This screen revealed that *S. cerevisiae* specifically responds to the *P. aeruginosa* QSM C12 and to none of the other compounds tested (Figure 1A). It has been shown before in *C. albicans* that the response to bacterial QSMs was limited to compounds with a 12-carbon backbone, similar in size to its native QSM sesquiterpene alcohol, farnesol (Shchepin et al., 2003). Although we did not observe a stress response upon exposure to farnesol, it has been demonstrated that farnesol affects *S. cerevisiae* in other ways, by inhibiting growth and inducing the generation of reactive oxygen species (ROS) (Machida et al., 1998, 1999). We further examined the specificity of the response by testing control compounds similar in structure to C12. The unnatural stereoisomer of C12, (*R*)-C12, induced a decreased response (Figure 1B), while C12-lactam, in which the oxygen of the lactone ring is replaced with a nitrogen, did not induce a stress response above control levels (Figure 1C). This indicates that the response to C12 is specific, and requires an intact lactone ring, a structural element shown to be crucial in the recognition of C12 in its native QS system in *P. aeruginosa*. When we further tested our compounds on a strain deleted in the transcription factors Msn4, the response was diminished, and in a double mutant strain in which both Msn4 and Msn2 were deleted, the response was abolished (Figure 1A). This data provides clear evidence that the response to C12 is Msn2/4 regulated, and to the best of our knowledge, is the first description of an Msn2/4-mediated stress response in yeast induced by a bacterial QSM.

In order to further explore the mechanism behind this stress response to C12, we collected mRNA microarray data. We compared four treatments: C12, hydrogen peroxide, C12-lactam, and DMSO. Hydrogen peroxide was chosen as a model for oxidative stress, as it is a well-characterized stress response and a common virulence mechanism for *P. aeruginosa*. Exposure to C12 resulted in a unique mRNA expression pattern, while the structurally related control compound, C12-lactam, yielded results similar to incubation with solvent alone (Figure 2). The response to incubation with C12 differs significantly from controls, but also from a previously described stress response to oxidative stress, which we replicated with hydrogen peroxide exposure. Several of the biochemical functions that are affected by up-and/or-down-regulated gene clusters upon C12 treatment overlap with those of the oxidative stress response. A number of genes involved in the Msn2/4 stress response pathways activated by hydrogen peroxide were similarly affected by exposure to C12, as expected for a Msn2/4-regulated response. For example, a number of antioxidants, including catalase T, transketolase, and glutaredoxin, were upregulated upon exposure to C12, with almost identical fold-change as in the response to hydrogen peroxide exposure (Hasan et al., 2002). Carbon metabolism was also strongly affected, with resources diverted to regenerate NADPH via slowdown of glycolysis and redirection to the pentose phosphate pathway (Estruch, 2000). Other pathways upregulated by both hydrogen peroxide and by C12 include genes involved in protein degradation, the synthesis of heat shock proteins and chaperones, while pathways involved in DNA replication were downregulated. Overall, more than 80% of the total genes affected by exposure to C12 were found to be shared in the response to hydrogen peroxide. However, substantially fewer genes in total were affected by C12 than by hydrogen peroxide treatment (806 vs. 2213), indicating that C12 may induce a more mild or specific stress response than oxidative stress. While the microarray results indicate a number of promising leads as described below, in future work these genes should be further validated by RT-PCR, and their relevance explored by using orthogonal methods such as single-gene knockout mutants.

Out of the remaining genes that were affected specifically by C12 and not by hydrogen peroxide, a number are associated with lipid synthesis and transport. One of the most striking genes found to be unique to the C12 response is *RSB1*, which was the most upregulated gene in this assay, with a 20-fold change compared to the control. *RSB1* encodes for a membranous protein, Rsb1p. The function of this protein has not been fully elucidated, but it has been implicated in the translocation of sphingolipids or similar compounds out of the cell, and may function as a flippase to incorporate sphingolipids into the membrane (Kihara and Igarashi, 2002). We have previously found in our work on bacterial-eukaryotic interactions that a mammalian sphingolipid, ceramide-1-phosphate, is involved in host immune response and pathogen recognition, sharing some of the same pathways shown to be affected by C12 in mammalian cells (Avni et al., 2010). Additionally, it has been suggested that C12 interferes with the integrity of mammalian cell membranes (Davis et al., 2010). The upregulation of *RSB1* in yeast is an intriguing finding adding to what is known about the effect of C12 on eukaryotic membranes, sphingolipids, and the innate immune response, and requires further study.

Another notable cluster we found to be affected in our microarray data is involved in iron homeostasis and transport. Previous work by Trejo-Hernandez et al. (2014) provides a very thorough analysis of differential protein expression in *C. albicans* in a mixed biofilm with *P. aeruginosa*. They conclude that iron deprivation is the driving force in the competition with the bacteria, showing that yeast mutants deficient in iron utilization were unable to compete with *P. aeruginosa* in mixed biofilms. The role of the pyoverdin siderophore produced by *Pseudomonas* sp. in competition between fungus and bacteria has already been demonstrated (Loper and Buyer, 1991; Harrison et al., 2008). It was shown that in mixed biofilms *P. aeruginosa* increases pyoverdin production in response to iron competition with *C. albicans* (Purschke et al., 2012). Our current data show that C12 has a significant effect on iron homeostasis in *S. cerevisiae*, causing downregulation of several genes involved in iron transport, assimilation, and homeostasis. In contrast, these same genes (*FTR1, FET3, SIT1, FRE1*) are upregulated upon exposure to hydrogen peroxide, indicating that this response pattern is distinct to C12.

In order to further investigate the effect of *P. aeruginosa* virulence factors toward *S. cerevisiae*, we performed a series of co-culture viability assays. We grew liquid co-cultures of the yeast with both the wild-type PAO1 and the C4/C12-deficient PAO-JP2, which produces fewer QS-regulated virulence factors. We found that in the liquid co-cultures, the viability of the yeast was significantly decreased by both strains of *P. aeruginosa* (Figure 3). We hypothesized that the observed effects on growth in liquid media are likely the result of direct competition between the yeast and the bacteria for nutrients and resources. Therefore, in order to isolate the effect of secondary metabolites, we grew *S. cerevisiae* in the presence of PAO1 or PAO-JP2 supernatant and found that exposure to PAO1 supernatant alone significantly inhibited yeast growth, while supernatant from the C4/C12-deficient PAO-JP2 did not, and in fact increased growth (Figure 3). Based on these data, it seems that various bacterial virulence factors or other secreted metabolites indeed play an important role in the inhibition of growth of *S. cerevisiae*, not only competition for resources, or other contact or protein mediated virulence effects. Since the effect was abolished in the PAO-JP2 supernatant, this indicates that these molecules are regulated by the C4 or C12 QS systems. Indeed, treatment with the JP2 supernatant resulted in increased growth, a surprising finding which indicates that perhaps other secreted molecules can also have a beneficial effect on the yeast, a point that requires further study.

Mild stress, such as that induced by C12 in this study, plays a crucial role in what is commonly known as the ESR (Gasch, 2007). It has been shown that if yeast is exposed to mild stress the cells undergo certain physiological changes that lead to overall higher stress tolerance to subsequent, more severe stress (Gasch and Werner-Washburne, 2002; Gasch, 2007). An ESR in response to *P. aeruginosa* C12 may be a good evolutionary adaptation to the intricate *Pseudomonas* QS system, as C12 may be an early indication of the induction of QS and the subsequent presence of QS-regulated virulence factors. One virulence factor in particular, pyocyanin, may be relevant, as it has the ability to oxidize and reduce other molecules and produce reactive oxygen species (ROS). Effects of ROS on cell metabolism are well documented in a variety of species. These include not only roles in apoptosis (programmed cell death) but also positive effects such as the induction of host defense genes (Conner et al., 2002; Rada and Leto, 2008) and mobilization of ion transport systems (Pottosin et al., 2014). In our current model, the ability of the yeast to sense C12 could be the first protective barrier against potential oxidative stress by ROS produced by pyocyanin (Tupe et al., 2015).

Therefore, we performed a rescue effect experiment in order to determine if a C12-induced ESR could indeed protect the yeast from a subsequent, stronger oxidative stress. *S. cerevisiae* cultures were pre-treated with C12 (mild stressor), and thereafter exposed to severe oxidative stress with the addition of H_2_O_2_ at a concentration which results in 50% cell death. We observed slight indications of an ESR-induced rescue effect, which appears to be strain-specific—a slight rescue effect was seen for wild-type strain BY4741, although the difference was not significant (Figure 4), while no effect was observed for wild-type strain W303.

Based on these data, we concluded that there is a C12-specific effect on *S. cerevisiae*. Therefore, in order to further elucidate the mechanism behind this response, we used our previously developed chemical proteomics approach to identify a eukaryotic receptor for this bacterial signaling molecule (Dubinsky et al., 2009, 2013; Rayo et al., 2021). We synthesized a diazirine-alkyne-tagged C12 analog to capture proteins undergoing interactions with the probe by activating the diazirine group and inducing covalent binding. The probe-protein complex can then be labeled via CuAAC chemistry to a fluorophore. Using this probe, we labeled several putative receptor proteins in a molecular weight range of ∼60–100 kDa, as well as some lower molecular weight proteins (Figure 5). We additionally analyzed samples using mass spectrometry, in order to identify proteins bound to the probe. One promising hit from the proteomics results was eIF-5A, a highly conserved eukaryotic protein previously linked to stress responses (Takeuchi et al., 2002; Li et al., 2010). These results represent preliminary, proof-of-concept experiments, and efforts in the lab to validate these results and explore potential yeast C12 receptors remain on-going.

Our studies indicate that the specific sensing of C12 by *S. cerevisiae* leads to a mild activation of its general stress response, which may enable it to prepare for more severe conditions—such as strong oxidative stress. Future work is needed to elucidate the mechanism by which this stress response is induced, as well as explore whether C12 affects S. cerevisiae in additional ways, such as the formation of pseudohyphae or fungal biofilms as has been documented for its native QS signals (Reynolds and Fink, 2001). Whether this directed activation of the stress response benefits the yeast, or *Pseudomonas aeruginosa*, or both, still remains to be resolved. A wealth of knowledge has been gained in recent decades on the importance of QS for bacteria in order to govern the production of various secondary metabolites; however, little is still known about the ability of other organisms to perceive such information on population density and use it to their benefit. This study offers a starting point to study a potential mechanism that may guide the interaction between *P. aeruginosa* and *S. cerevisiae* and may reflect the relationships between bacterial and fungal species that compete and coexist in other environmental niches.

## Data Availability Statement

The datasets presented in this study can be found in online repositories. The names of the repository/repositories and accession number(s) can be found below: https://www.ebi.ac.uk/arrayexpress/, E-MTAB-4802.

## Author Contributions

AD, AA, and MM conceived and designed the experiments. AD, RG, LD, RD, JR, and AH performed the experiments. AD, AH, RG, AA, and MM analyzed the data. LD, RD, PK, and JR contributed reagents, materials, and analysis tools. AD, RG, AA, and MM wrote the manuscript. All authors contributed to the article and approved the submitted version.

## Conflict of Interest

The authors declare that the research was conducted in the absence of any commercial or financial relationships that could be construed as a potential conflict of interest.

## Publisher’s Note

All claims expressed in this article are solely those of the authors and do not necessarily represent those of their affiliated organizations, or those of the publisher, the editors and the reviewers. Any product that may be evaluated in this article, or claim that may be made by its manufacturer, is not guaranteed or endorsed by the publisher.

## Footnotes

1
http://pathway.yeastgenome.org/overviewsWeb/celOv.shtml

2
http://funspec.med.utoronto.ca/
