# Supplementary material for: A Bacterial Quorum Sensing Molecule Elicits a General Stress Response in Saccharomyces cerevisiae
Source: Front Microbiol. 2021 Sep 16;12:632658. doi: 10.3389/fmicb.2021.632658 (PMC8481950; doi:10.3389/fmicb.2021.632658)
Supplement: Supplementary file 1 [file Image_1.pdf]

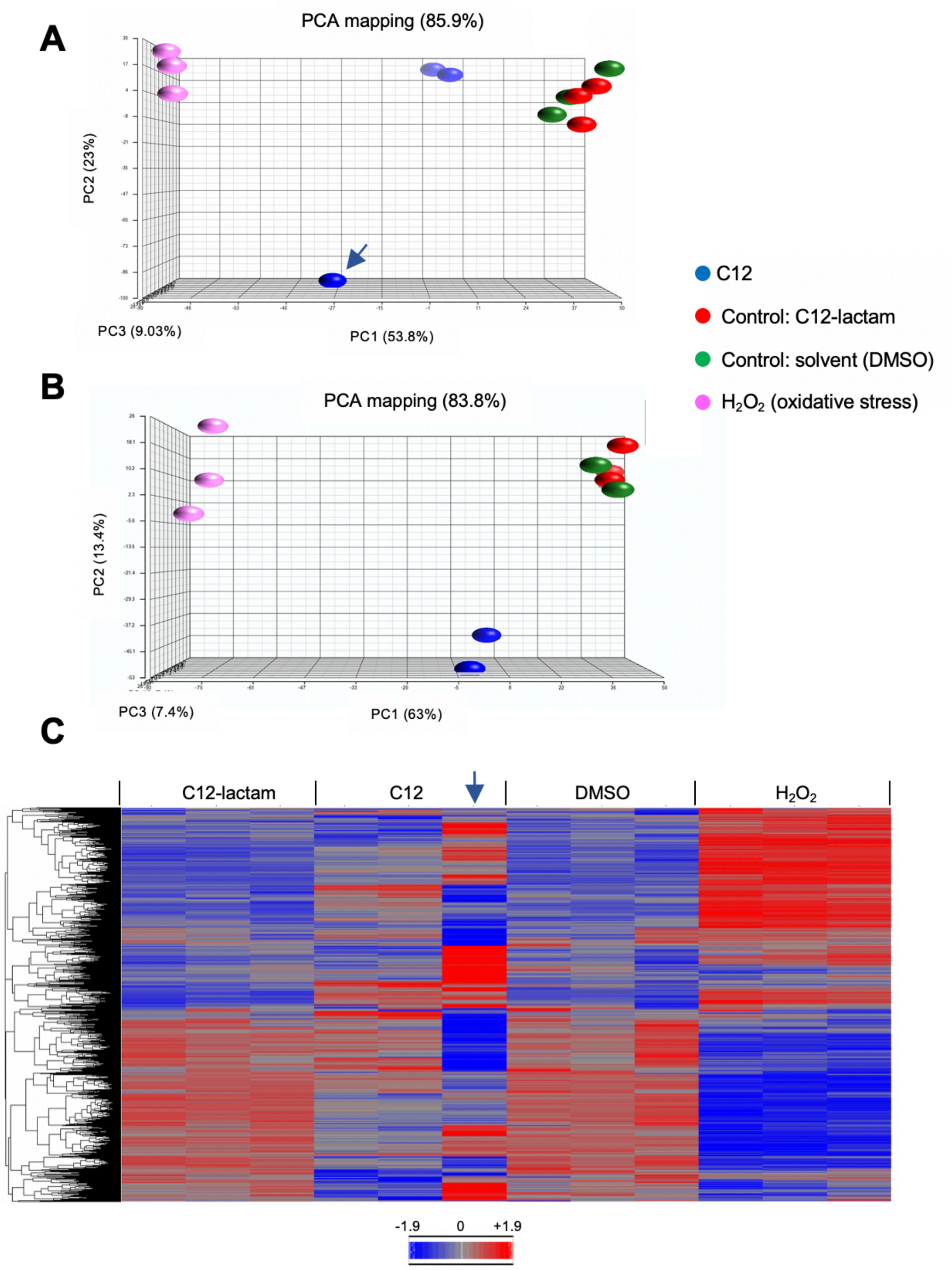

Fig. S1. Initial microarray analysis. (**A**) PCA analysis of the samples showed similarity between the two control treatments (solvent control and C12-lactam). The hydrogen peroxide treatment clustered distinctly from the C12 treatment. Of the C12 samples, one sample from the biological triplicates appeared as an outlier in the PCA analysis (marked with a blue arrow), and was therefore removed from subsequent analysis, as shown in (**B**). (**C**). Hierarchal clustering using Pearson dissimilarity metric of all signals (filtered for max signal over 5, after Z-scoring). The outlier sample removed from analysis is marked with a blue arrow.
